# A Facile Two-Step High-Throughput Screening Strategy of Advanced MOFs for Separating Argon from Air

**DOI:** 10.3390/nano15060412

**Published:** 2025-03-07

**Authors:** Xiaoyi Xu, Bingru Xin, Zhongde Dai, Chong Liu, Li Zhou, Xu Ji, Yiyang Dai

**Affiliations:** 1 School of Chemical Engineering, Sichuan University, Chengdu 610065, China; xuxu_xxy@163.com (X.X.); xinbingru@gmail.com (B.X.); liuchong@scu.edu.cn (C.L.); chezli@scu.edu.cn (L.Z.); 2 School of Carbon Neutrality Future Technology, Sichuan University, Chengdu 610065, China; zhong-de.dai@scu.edu.cn

**Keywords:** Grand Canonical Monte Carlo simulation, metal–organic frameworks, argon, pressure swing adsorption, gas separation

## Abstract

Metal–organic frameworks (MOFs) based on the pressure swing adsorption (PSA) process show great promise in separating argon from air. As research burgeons, the number of MOFs has grown exponentially, rendering the experimental identification of materials with significant gas separation potential impractical. This study introduced a high-throughput screening through a two-step strategy based on structure–property relationships, which leveraged Grand Canonical Monte Carlo (GCMC) simulations, to swiftly and precisely identify high-performance MOF adsorbents capable of separating argon from air among a vast array of MOFs. Compared to traditional approaches for material development and screening, this method significantly reduced both experimental and computational resource requirements. This research pre-screened 12,020 experimental MOFs from a computationally ready experimental MOF (CoRE MOF) database down to 7328 and then selected 4083 promising candidates through structure–performance correlation. These MOFs underwent GCMC simulation assessments, showing superior adsorption performance to traditional molecular sieves. In addition, an in-depth discussion was conducted on the structural characteristics and metal atoms among the best-performing MOFs, as well as the effects of temperature, pressure, and real gas conditions on their adsorption properties. This work provides a new direction for synthesizing next-generation MOFs for efficient argon separation in labs, contributing to energy conservation and consumption reduction in the production of high-purity argon gas.

## 1. Introduction

Argon is the most abundant inert gas in the air (about 0.932%) [1]. It is characterized by being colorless, odorless, and non-toxic, with extremely low chemical reactivity [2], and has a wide range of applications in industry, medicine, environmental protection, mining, geology, manufacturing, and other fields. For example, argon plays an important role in semiconductor manufacturing and modification processes [3], significantly improves the efficiency of hydrogen production through methanol sono-pyrolysis decomposition [4,5], and can be used as protective gas in welding processes to prevent welding defects and oxidation [6]. In materials science, nanofilms are frequently subjected to modification processes utilizing argon environments or argon ion bombardment systems to tailor their properties [7,8]. In the aerospace field, argon is widely used as a coolant for spacecraft [9]. In the food production process, argon-modified atmosphere packaging can achieve the affect of maintaining product freshness and extending shelf life [10]. In addition, argon gas knives are used in clinical surgical treatment [11,12], and argon lasers are used for treating various eye diseases and skin conditions [13]. A more common use of argon gas in daily life is in the manufacture of light bulbs [14]. In summary, the above applications demonstrate the versatility and importance of argon in industrial production.

Currently, the primary method for producing argon is air separation, which involves low-temperature distillation from liquid air, followed by drying and cooling to achieve large-scale argon separation based on the different boiling points of each component [15,16]. Moreover, the physical properties of pure oxygen and pure argon are very similar, which results in a high reflux ratio for rectification [17]. Typically, the height of a conventional super-tower needs to exceed 60 m to obtain high-purity argon, making the main challenges of this method high energy consumption, high cost, complex production processes [18,19], and a certain degree of risks. Pressure swing adsorption (PSA) separation [20] is an energy-efficient, easy-to-operate, low-regeneration-cost, environmentally friendly, and highly reliable treatment technology, which may offer a new solution to the aforementioned issues for efficiently separating argon from air. In recent years, some preliminary schemes have been proposed in the literature to achieve the production of high-concentration argon gas through PSA separation [21,22]. As referenced in Saburo Hayashi’s research [23,24], a five-stage dynamic control PSA method has been proposed to separate argon from air. Two different adsorbents (zeolites and a molecular sieve) are packed in a single adsorption column to sequentially absorb nitrogen and oxygen, with the maximum pressure swing configuration set between 0.13 bar and 3.5 bar, thereby increasing the concentration of argon after multiple cycles of operation. The challenge with this method is the difficulty of simultaneously achieving optimal adsorption conditions for different adsorbents within the column, leading to a significant increase in energy and material consumption. Consequently, discovering an adsorbent capable of efficiently capturing both nitrogen and oxygen simultaneously would effectively address the aforementioned challenges and difficulties.

Metal–organic frameworks (MOFs) [25], a novel class of nanoporous materials, are constructed through the self-assembly of metal ions or clusters and organic ligands, forming a periodic mesh topology. These materials are characterized by their large surface areas, high porosity, tunable pore sizes, strong functionality, and significant adsorption activity. In recent years, MOFs have garnered extensive attention and have been widely applied in the adsorption and separation of gases, showcasing immense potential and an enticing future prospect [26,27,28]. To date, tens of thousands of MOFs have been synthesized, such as the computationally ready experimental MOF database (CoRE MOF 2019) constructed by the Snurr group [29], which includes 12,020 MOFs. The diversity, quantity, and complexity of these materials make it impractical to rely solely on the traditional material discovery processes, which involve synthesis, structural characterization, and adsorption experiments of each MOF, akin to finding a needle in a haystack. With the booming development of computational chemistry and the substantial increase in computational power, high-throughput computational screening (HTCS) methods based on molecular simulations have become a powerful tool to accelerate material discovery for various applications [28,30,31,32]. Existing research on Ar adsorption or separation has primarily focused on zeolites, molecular sieves [22,33,34], or certain specific types of MOF materials [35] compared to the number of MOFs, with only ordinary results achieved. There are currently no reports in the literature on high-throughput screening studies of MOF adsorbents for separating argon in air, and potential high-performing MOF adsorbent candidates may remain undiscovered.

The computational cost and time required for molecular simulation calculations on the entire dataset would be substantial. This study proposes a high-throughput hierarchical screening strategy based on Grand Canonical Monte Carlo (GCMC) simulations to efficiently identify MOF adsorbents with high argon–air separation performance from the CoRE MOF database, significantly reducing computing costs and enhancing the material screening process. Initially, materials were pre-screened based on the kinetic diameter of the adsorbed gases, excluding MOFs in the database that clearly do not meet the requirements. Subsequently, the pre-screened databases were randomly sampled, and the corresponding adsorption properties were obtained through simulation calculation. The remaining MOF adsorbents were screened based on structure–property relationships. Then, rigorous molecular simulations were used again to evaluate the adsorption performance of the MOFs within the optimal structure range, including adsorption selectivity, working capacity, the adsorbent performance score for argon, and regenerability. Finally, the relationship between the structure or metal type of the top 10 MOF adsorbents and their adsorption performance, as well as the impact of relevant factors such as temperature, pressure, and real gas conditions on the adsorption separation performance, were studied.

## 2. Methods

**Figure 1:**
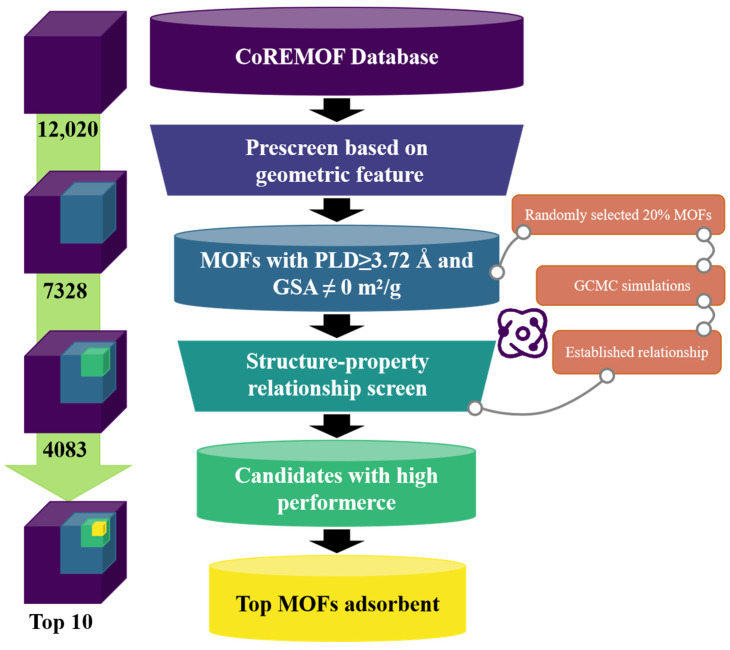
Workflow for high-throughput screening of MOF adsorbents designed to separate argon from air.

This section provides a detailed overview of the high-throughput two-step screening strategy based on GCMC simulations. This strategy was employed for the screening of high-performance MOF adsorbents designed to separate argon gas from air. The workflow is illustrated in Figure 1.

### 2.1. MOF Database and Descriptors

This study employed the comprehensive CoRE MOF 2019 database, built by Snurr et al., which comprises a rich collection of 12,020 structurally diverse MOFs represented by their Crystallographic Information Files (CIFs). To thoroughly elucidate the characteristics of the MOFs, the open-source software Zeo++ 0.3 [36] was utilized to compute six key descriptors that are widely recognized and easily calculable in the field of gas separation. These descriptors, which include the largest cavity diameter (LCD), pore-limiting diameter (PLD), geometric surface area (GSA), volume surface area (VSA), porosity (Φ), and density (ρ) [37,38,39], serve to accurately depict the geometry and dimensions of the internal channels. A degree of intercorrelation between the six indicators is also illustrated in Figure S1. The abstract information encoded in CIF files was effectively translated, such as atomic coordinates and cell parameters, into quantifiable features, thus generating a detailed numerical profile for each MOF. Notably, the estimation of Φ was conducted using the RASPA 2.0 code [40], while the remaining descriptors were computed through the zeo++ software. The GSA and VSA were determined using nitrogen molecules with a kinetic diameter of 3.64 Å [41] as spherical probe molecules [42]. In addition to their geometric characterization, we detailed the chemical properties by specifying the metal type, quantity, and the presence of open metal sites (OMSs) for each MOF. These details were obtained through an OMS detection algorithm [43]. Furthermore, we analyzed the proportion of OMS existence in MOFs sharing the same metal ligands, which provides a comprehensive discussion on how metal ligands and OMSs contribute to the adsorption performance of MOFs in the Results and Discussion section.

### 2.2. Grand Canonical Monte Carlo (GCMC) Simulation

Leveraging the RASPA 2.0 software, GCMC simulations were employed to investigate the adsorption behavior of the PSA process for an ideal ternary air mixture (comprising argon, oxygen, and nitrogen at mole fractions of 0.01:0.21:0.78) in MOFs. The simulations were conducted at 298 K, with adsorption pressures set at 1 bar and desorption pressures set at 0.1 bar. The Peng–Robinson equation of state was utilized to convert pressure to fugacity. Consequently, the key performance metrics for the Ar/O_2_/N_2_ adsorption separation process in MOFs were calculated.

At the microscale, the adsorption behavior of MOFs in gas is primarily characterized by the interactions between MOFs and gas molecules. For computational efficiency, the MOF frameworks were treated as rigid, and intramolecular interactions within the MOF structure were neglected. Consequently, the total energy of the adsorption system (U) was mainly composed of two parts: the interaction between adsorbate molecules and the MOF framework and the interactions among adsorbate molecules themselves [44,45].

(1) $$U=U_{LJ}+U_{elec},$$

(2) $$U_{LJ}=4\varepsilon_{ij}[{\left(\frac{\sigma_{ij}}{r_{ij}}\right)}^{12}-(\frac{\sigma_{ij}}{r_{ij}}{)}^{6}],$$

(3) $$U_{elec}=\frac{1}{4\pi \varepsilon_{0}}\times \frac{q_{i}q_{j}}{r_{ij}},$$

In Formula (1), $U_{LJ}$ represents short-range Van der Waals interactions and $U_{elec}$ represents long-range electrostatic interactions, which are calculated through the Ewald summation method. In Formulas (2) and (3), $\varepsilon_{ij},\;\sigma_{ij}\;and\;r_{ij}$ are the potential well depth, the Lennard–Jones radius, and the distance between atoms i and j, respectively; $q_{i}$ and $q_{j}$ are the partial charge of atom i and j; and $\varepsilon_{0}$ is the dielectric constant (8.854 × 10^−12^ F/m) [46].

The atomic charges were expeditiously assigned using the Qeq method integrated into the RASPA 2.0 software. The Lennard–Jones (LJ) potential parameters for framework atoms were sourced from the Universal Force Field (UFF) [47]. The LJ cutoff distance was set to 13 Å [48], which means that the unit cell dimensions along each of three spatial directions must be extended to at least 26 Å, with periodic boundary conditions applied. The specific Lennard–Jones parameters of the MOFs and each gas molecule are provided in Tables S1 and S2 [49]. The interaction parameters between two different atoms are usually calculated by the Lorentz–Berthelot mixed rule.

(4) $$\sigma_{ij}=(\frac{\sigma_{i}+\sigma_{j}}{2}),$$

(5) $$\varepsilon_{ij}=\sqrt{\varepsilon_{i}\varepsilon_{j}},$$

In Formulas (4) and (5), $\sigma_{i}$ and $\sigma_{j}$ are the potential well depth of atom i and j, respectively; $\varepsilon_{i}$ and $\varepsilon_{j}$ is the collision distance of atom i and j, respectively.

For each MOF, the GCMC simulation computation was carried out in 20,000 cycles, with the initial 10,000 cycles allocated for system equilibrium and the remaining 10,000 cycles utilized for computing the average thermodynamic properties from the sampled data. Each cycle considered a total of n times different types of equally probable Monte Carlo (MC) test moves, where n is the number of adsorbent molecules. These moves included random translation, random rotation, and random reinsertion [50,51]. For comprehensive validation of the force fields and parameters’ accuracy, applicability, and reliability, please refer to Figure S2 [52,53]. The performance metrics of the MOF adsorbent, including working capacity (∆N), selectivity (S), regeneration performance (R%), the adsorbent performance score (APS) as a balance between working capacity and selectivity, and the adsorbent performance score for separating argon from air (APSA), were calculated using the following formulas:

(6) $$∆N_{i}={∆N}_{i}^{ads}-{∆N}_{i}^{des},\;(i\mathrm{represents}\mathrm{different}\mathrm{adsorbates})$$

(7) $$S_{N_{2}/(O_{2}+\mathrm{Ar})}=\frac{N_{N_{2}}^{ads}/(N_{Ar}^{ads}+N_{O_{2}}^{ads})}{f_{N_{2}}/(f_{Ar}+f_{O_{2}})},$$

(8) $$S_{O_{2}/(N_{2}+\mathrm{Ar})}=\frac{N_{O_{2}}^{ads}/(N_{Ar}^{ads}+N_{N_{2}}^{ads})}{f_{O_{2}}/(f_{Ar}+f_{N_{2}})},$$

(9) $${APS}_{\left(N_{2}\right)}=∆N_{N_{2}}\times S_{N_{2}/(O_{2}+\mathrm{Ar})},$$

(10) $${APS}_{\left(O_{2}\right)}=∆N_{O_{2}}\times S_{O_{2}/(N_{2}+\mathrm{Ar})},$$

(11) $$R\%(\alpha )=\frac{∆N_{Ar}}{{∆N}_{\alpha }^{ads}}\times 100\;(\alpha \mathrm{stands}\;\mathrm{for}\;\mathrm{oxygen}\;\mathrm{and}\;\mathrm{nitrogen})$$

(12) $$APSA={APS}_{\left(N_{2}\right)}\times {APS}_{\left(O_{2}\right)},$$

### 2.3. Two-Step Screening Strategy

#### 2.3.1. Pre-Screening

Given that not all MOFs within the CoRE MOF database are suitable for capturing nitrogen or oxygen, it would be futile to apply rigorous molecular simulations to every MOF, which would unnecessarily consume substantial computational resources and time. To optimize the process, a preliminary geometrical analysis was conducted prior to the large-scale GCMC simulations. This pre-screening step eliminated MOFs with a pore-limiting diameter (PLD) less than 0.364 nm (corresponding to the kinetic diameter of nitrogen) [54] and those with a geometric surface area (GSA) of zero, as these MOFs were obviously unable to adsorb nitrogen. Following this rigorous pre-screening step, 7328 MOFs remained in the database for further investigation.

#### 2.3.2. Screening Based on Structure–Property Relationship

**Figure 2:**
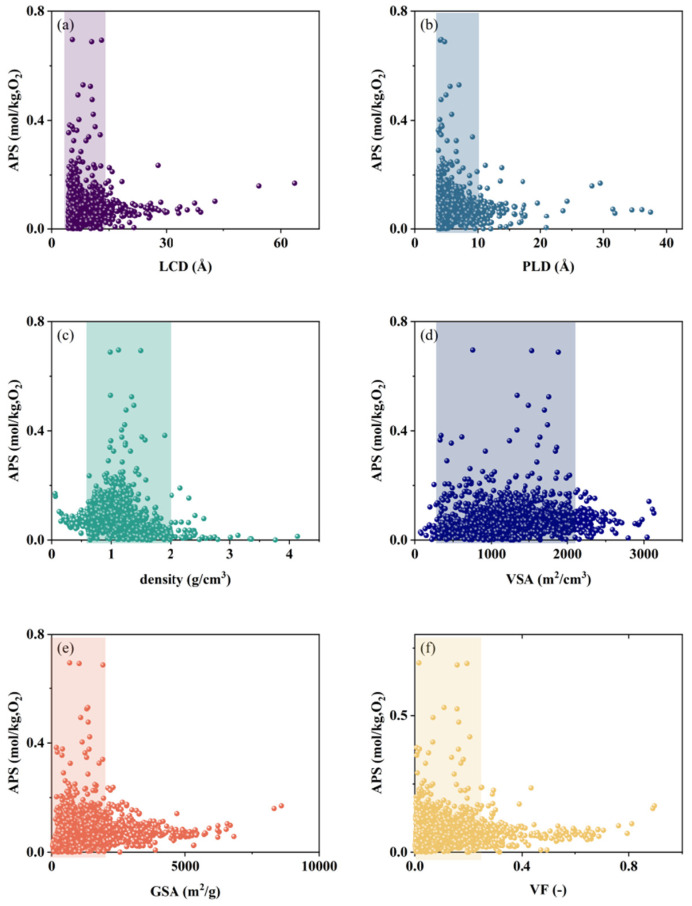
The structure–property relationship based on oxygen as the adsorbent; the structure–property relationship based on nitrogen as the adsorbent is described in Figure S6. (**a**) LCD; (**b**) PLD; (**c**) density; (**d**) VSA; (**e**) GSA; (**f**) VF.

A random subset comprising 20% of the MOFs from the pre-screening database was selected for detailed GCMC simulations. These simulations aimed to model the adsorption behaviors in a ternary gas mixture (N_2_/O_2_/Ar). The adsorption properties of the selected MOFs, including APS, S, ∆N, and R%, were obtained. As depicted in Figure 2, it can be observed that the performance of the adsorbent is significantly superior within specific intervals. Then, the optimal structural intervals for adsorption with N_2_ and O_2_ as the target gases were identified, respectively. Given the study’s objective of finding adsorbents capable of efficiently adsorbing both oxygen and nitrogen to facilitate argon separation, the targeted optimal geometric structure range was defined as the intersection of the two identified intervals. Table 1 lists the specific ranges of these optimal geometric intervals, which are likely to contain the target MOF adsorbents.

**Figure 3:**
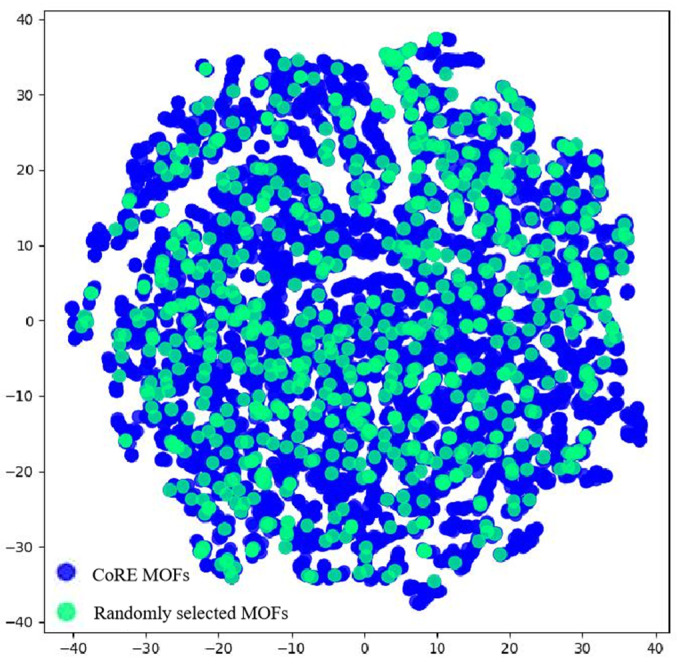
t-SNE algorithm visualized the sampling points and the pre-screening database.

To ensure the representativeness of the sampling, the t-SNE algorithm [55] was utilized to visualize the position of each MOF in geometric space. The six geometric structural descriptors of MOFs calculated in the previous section served as the defining features of MOFs in geometric space. By applying the t-SNE algorithm for dimensionality reduction, the similarities in geometric structure between the sampling points and the pre-screening database were revealed. The parameters associated with the t-SNE algorithm were comprehensively listed in Table S6. As demonstrated in Figure 3, the randomly selected sample points were uniformly and sensibly dispersed throughout the data space, suggesting that the structure–property relationships derived from the subset of MOFs were generalizable to the broader MOF domain.

#### 2.3.3. Top MOFs Selection

The MOFs located in the target optimal structural interval were identified as potential candidate adsorbents. Comprehensive GCMC simulations were conducted on these candidates, with nitrogen and oxygen serving as the target adsorbates, respectively. The candidates were ranked based on the magnitude of their APSA. Specific thresholds for the APS of nitrogen and oxygen were established, set at 1.036 and 0.149, respectively. These thresholds corresponded to the top 10% values of the APS among the MOFs when either N_2_ or O_2_ was utilized as the adsorbate.

If a particular MOF has a high APSA ranking and its APS values for both nitrogen and oxygen are not lower than the respective thresholds, it indicates that this MOF adsorbent can efficiently adsorb both N_2_ and O_2._ Such MOFs are considered high-performance adsorbents for the separation of argon from air. Taking into account that adsorbents with excessively low regeneration performance hold little industrial significance, this study selected ten MOF adsorbents with an R% greater than 80% and the highest APSA ranking. Further structural and chemical analyses were then conducted on these high-performance adsorbents. Considering the variability in temperatures and pressures in actual industrial production, this study also delved into the effects of these parameters on adsorption efficacy.

## 3. Results and Discussion

### 3.1. Separation Performances of MOFs

**Figure 4:**
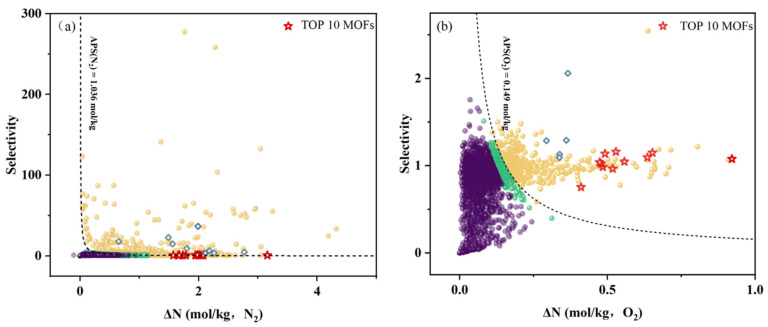
The relationship between the working capacity and selectivity of MOF adsorbents within the optimal structural range. The yellow, green, and purple spheres separately refer to MOFs with top 10%, top 20%, and bottom 80% APSs for nitrogen or oxygen as the target adsorbate. The grey boxes refer to MOFs with high adsorption capabilities for nitrogen or oxygen. (**a**) The target adsorbate is nitrogen; (**b**) the target adsorbate is oxygen.

Figure 4 delineated the GCMC simulation outcomes for 4083 MOFs situated within the target optimal structural interval, mapping out the correlation between their adsorption working capacity and selectivity. This analysis is pivotal for pinpointing high-performance MOF adsorbents tailored for argon separation from air. As depicted in Figure 4a, spheres represent individual MOF adsorbent materials, with color-coding corresponding to their respective APS for nitrogen adsorption. Spheres in yellow highlight MOFs with the top 10% APSs, indicating the highest adsorption capacity for nitrogen. Those in green denote MOFs with the APS ranking falling between 10% and 20%, indicating a more moderate adsorption capacity for nitrogen. Spheres in purple represent MOFs whose APS ranking does not fall within the top 20%, indicating that MOFs of this category are ill-suited for the adsorption of nitrogen. Notably, the MOFs most likely to serve as effective argon–air separation adsorbents are clustered in the yellow circle region of both figures. Figure 4b, as presented, is analogous to Figure 4a, with the primary difference being that its target adsorbate is oxygen.

**Figure 5:**
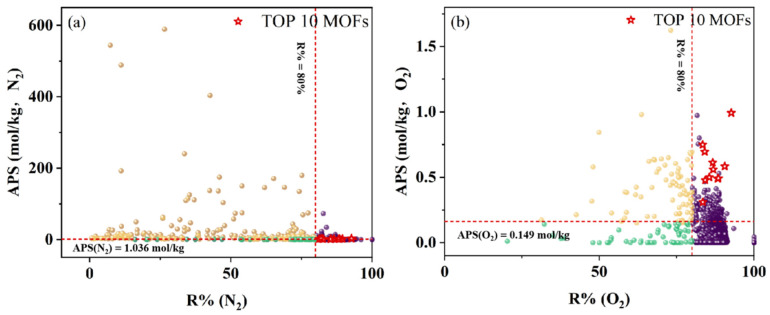
The relationship between the APS and R% of MOF adsorbents. The purple spheres refer to MOFs with R% > 80%, the yellow spheres refer to MOFs with R% < 80% and the top 10% APSs, and the green spheres refer to MOFs with R% < 80% and bottom 90% APSs for nitrogen or oxygen as the target adsorbate. (**a**) The target adsorbate is nitrogen; (**b**) the target adsorbate is oxygen.

However, it is crucial to recognize that relying solely on the APS for selecting high-performance MOF adsorbents is insufficient. As illustrated in Figure 5, certain MOFs may exhibit elevated APSs alongside a suboptimal R%. In this study, MOFs with R% values below 80% were excluded. Such MOFs, despite their high APSs, may not be suitable for industrial applications [56]. Consequently, the ideal MOF adsorbents for argon–air separation are those that excel in the APSA and APS for nitrogen and oxygen (when each is used as the target adsorbate) and R%.

Taking Figure 4a as an example, the MOFs situated in the upper right corner of the graph demonstrated superior performance in the mixed gas system with N_2_ as the adsorbed molecule. But these MOFs may exhibit subpar performance in the same system when O_2_ is the adsorbed molecule, or they may have poor regeneration efficiency. These factors explained why some yellow-circled MOFs located in the upper right corner of Figure 4a were not selected as the top 10 MOFs. By the way, the gray boxes in Figure 4 delineate a subset of MOFs that exhibit commendable adsorption capabilities for N_2_ in Figure 4a and for O_2_ in Figure 4b. While these materials may not be ideal for argon separation from air, their properties render them promising candidates for applications in nitrogen adsorption or nitrogen–oxygen separation.

This research concludes with the identification of 10 prospective MOF adsorbents for separating argon from air, as distinctly marked by the red five-pointed stars within Figure 4 and Figure 5. Their detailed performance metrics and parameters are summarized in Table 2.

### 3.2. Metals and OMSs

**Figure 6:**
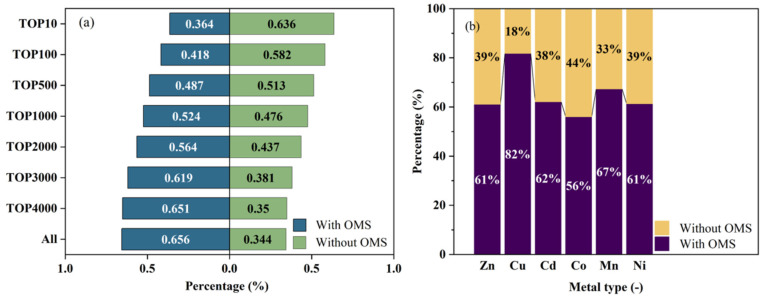
(**a**) Proportion of OMSs in top MOFs; (**b**) proportion of OMSs in MOFs of the same metal.

This research scrutinized the existence of open metal sites (OMSs) and the type of metal ions or clusters in MOFs [57], utilizing an OMS detection algorithm [43]. It further examined the impact of these features on the adsorptive behavior of MOFs, specifically within the framework of argon–air separation. Initially, an analysis was conducted to explore the correlation between the adsorption properties of randomly selected 20% MOFs in the pre-screening database and the presence of OMSs. This was achieved by juxtaposing the APS values of MOFs with and without OMSs, with the results graphically depicted in Figure S7 for a comprehensive understanding. The result revealed that the presence of OMSs did not significantly enhance the APS values. Accordingly, the presence of OMSs was not incorporated as a selection criterion in the screening stage. Subsequently, the distribution of MOFs containing OMSs across various groups was examined. As depicted in Figure 6a, there was an observable trend where the proportion of MOFs with OMSs diminishes alongside the progressive enrichment of the top MOFs. This observation suggested that the presence of OMSs exerted a modicum of inhibitory influence on the efficacy of adsorbents designed for the separation of argon from air.

**Figure 7:**
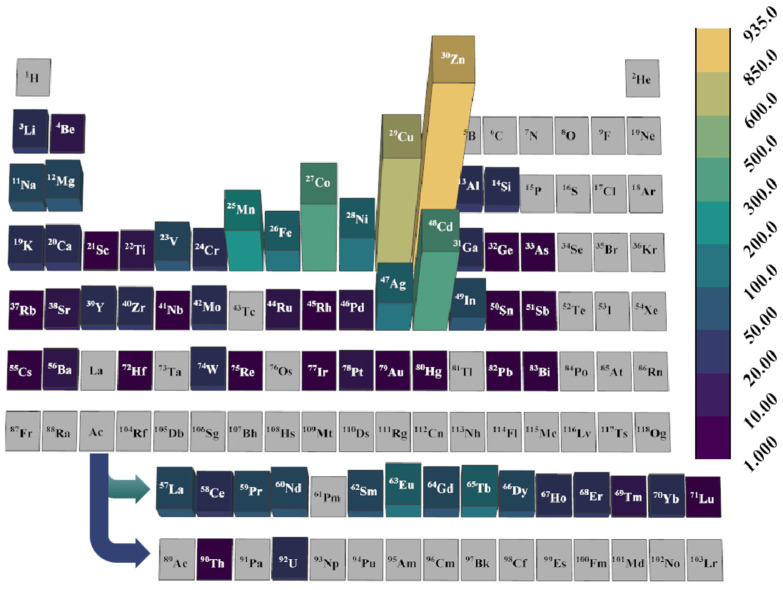
Metal ligand types and numbers of all candidate MOFs.

Figure 7 illustrates the type of metal ions or clusters present in all candidate MOFs within the optimal structural interval for the high-performance separating adsorption of argon from air. The changes in color and column height reflected the distribution and frequency of each metal. Among the candidate MOFs, zinc (Zn) is identified as the most prevalent metal, present in 935 MOFs. Other notable metals, in descending order of frequency, include copper (Cu), cadmium (Cd), cobalt (Co), manganese (Mn), nickel (Ni), and silver (Ag). Detailed information about the concentration of OMSs and metal types is provided in Table S3.

Further analysis focused on the existence of OMSs in MOFs with a higher count of the metal. MOFs incorporating copper (Cu) as a metal ligand were observed to have the highest proportion of OMS presence, whereas those with a cobalt (Co) metal ligand exhibited the lowest frequency of OMS occurrence. This finding revealed the influence of specific metal ions or clusters on the formation and prevalence of OMSs within the MOF structures (see Figure 6b).

### 3.3. Comparison with Molecular Sieve Separation Data

**Figure 8:**
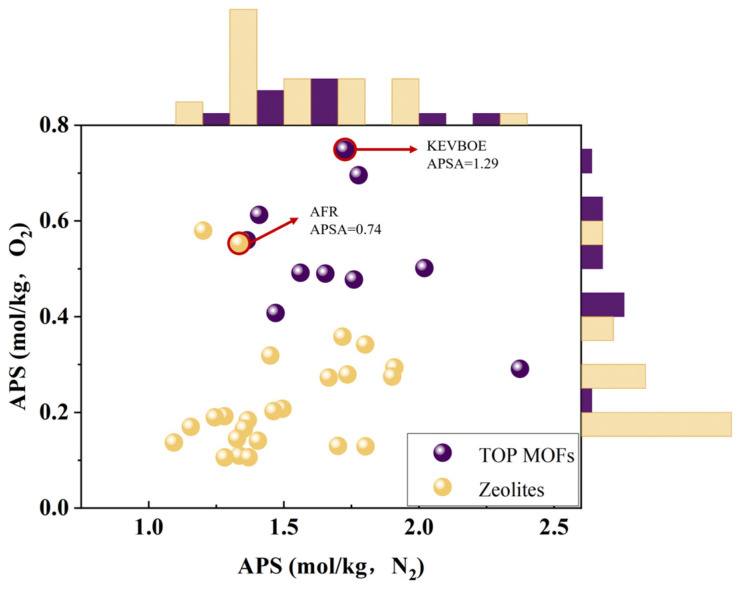
Comparison of adsorption properties of top MOFs and zeolites.

At present, most adsorbents used in the separation of argon from air by the PSA process are still focused on zeolite or molecular sieve materials. To verify the validity of this work and the high performance of the selected MOF adsorbents, this study simulated the adsorption behaviors of all zeolites with known crystal structures (a total of 214) in a ternary gas mixture (Ar:N_2_:O_2_) system. The structural information on all of the zeolite framework types was derived from the Database of Zeolite Structures approved by the Structure Commission of the International Zeolite Association (IZA-SC) [58]. Adsorption was conducted at a pressure of 1 bar, desorption at 0.1 bar, and a temperature of 298 K. Ultimately, only 24 zeolite materials with an ${APS}_{\left(N_{2}\right)}$ and ${APS}_{\left(O_{2}\right)}$ above the threshold and R% greater than 80% were retained. When compared with the top MOFs identified through this investigation, a comparative analysis revealed that, in general, the adsorption performance of the top MOFs tended to outperform that of the zeolite adsorbents. Specifically, the APSA of the best-performing MOF (KEVBOE) was 1.29, which was 1.75 times higher than that of the best-performing zeolite (AFR). The findings from this study clearly demonstrate that MOFs possess significant potential and exhibit formidable capabilities as adsorbents for the separation of argon from air (see Figure 8).

### 3.4. Analysis of Temperature, Pressure, and Compatibility

**Figure 9:**
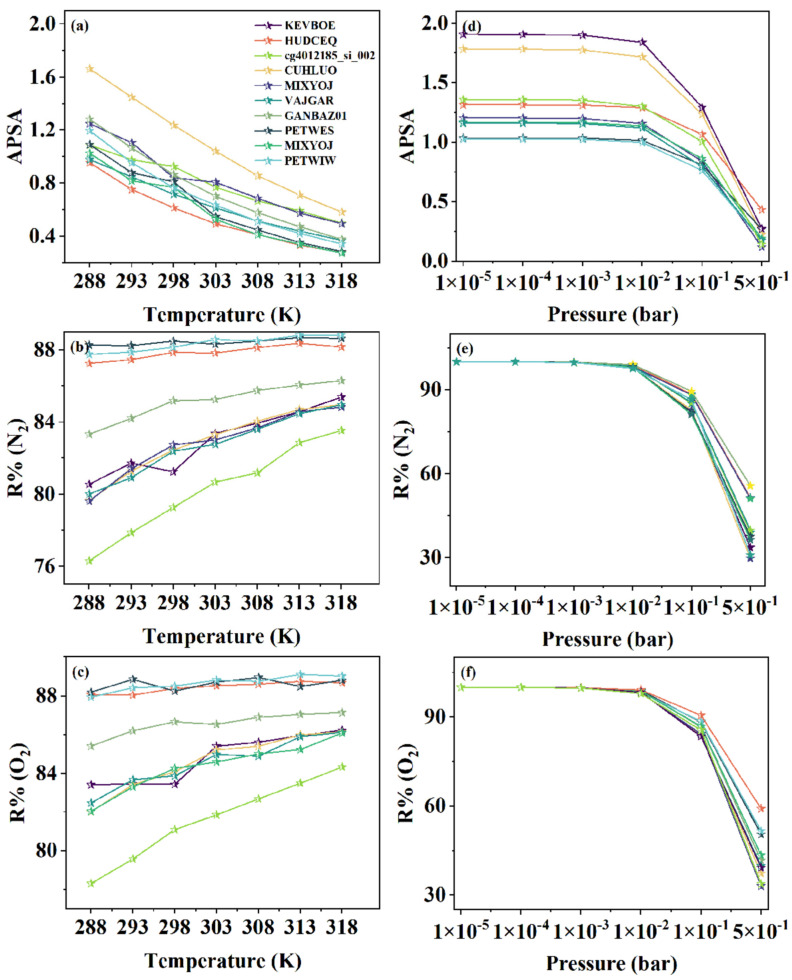
The impact of temperature and desorption pressure on the adsorbent’s APSA and regenerability. With the changes in temperature: (**a**) the variation of the APSA; (**b**) the variation of the R% for nitrogen as the target adsorbate; (**c**) the variation of the R% for oxygen as the target adsorbate. With the changes in pressure: (**d**) the variation of the APSA; (**e**) the variation of the R% for nitrogen as the target adsorbate; (**f**) the variation of the R% for oxygen as the target adsorbate.

For adsorption processes, temperature is a critical and practical parameter that significantly influences the efficacy of adsorbents. In this study, the adsorption equilibrium of the top 10 MOFs was modeled across a range of temperatures using GCMC simulations, with the resulting data elegantly portrayed in Figure 9. The data suggested a consistent pattern for both nitrogen and oxygen as target adsorbates: as the temperature improved, the adsorbent’s regenerative capability increased progressively, while the APSA values decreased significantly. After a thorough evaluation of these metrics, the research determined that a simulation temperature of 298 K was the most reasonable choice. This temperature ensured that R% remained robustly above 80% while also delivering an impressively high APS, thereby balancing regeneration efficiency and adsorption performance.

Figure 9 delineates the effects of different desorption pressures on the adsorbent performance, with the adsorption pressure held constant at 1 bar and the desorption pressure varying from 10^−5^ to 0.5 bar. The data revealed that as the desorption pressure decreased, there was a marked improvement in both the APSA and R%. When the desorption pressure reached approximately 0.01 bar, the various adsorption performance metrics plateaued, indicating no further significant enhancement in the adsorption performance with lower desorption pressure. Selectivity, as defined, is inherently linked to the adsorption phase alone and remains unaffected by the desorption phase. Therefore, changes in desorption pressure had no bearing on selectivity. This trend can be explained by the correlation between energy input and separation efficiency. A lower desorption pressure results in a higher vacuum, necessitating more energy input. This heightened energy input is directly correlated with enhanced separation efficiency of the argon–air mixture. Yet, while lower desorption pressures may enhance the separation efficacy, they also translate into higher economic costs and increased operational difficulty, highlighting a delicate balance between argon recovery efficiency and economic feasibility within the gas system. The top 10 MOFs identified by this study effectively reconcile adsorption efficacy with economic viability within a desorption pressure range of 0.02 to 0.1 bar.

Deviation from this range, either by further reduction or increasing the desorption pressure, could escalate economic costs or diminish adsorption performance. Moreover, continuous desorption at high pressures may compromise the structural stability of the adsorbents, potentially leading to degradation in adsorption stability.

### 3.5. The Performance of the Adsorbent Under Real Gas Conditions

In previous research, the gas mixture was modeled as an ideal ternary blend of argon, oxygen, and nitrogen, with a composition ratio of 0.01:0.21:0.78. Nevertheless, in practical industrial air separation applications, the presence of other gas impurities is unavoidable, notably carbon dioxide (CO_2_) and water (H_2_O). Consequently, an assessment was carried out under more realistic gas circumstances for the top 10 MOFs, with a composition of Ar:N_2_:O_2_:CO_2_:H_2_O = 0.0094:0.78:0.21:0.0004:0.0002. The H_2_O molecules were modeled using the TIP4P/2005 model, which consists of a Lennard–Jones site for the oxygen atom and three charge sites [59]. The relevant parameters for the GCMC simulation are detailed in Table S2. Table 3 illustrates the adsorption capabilities of the top 10 MOFs identified in this study. Under these more authentic gas conditions, the performance of the adsorbents in isolating argon from air has modestly declined. Nonetheless, the adsorbents still operate at a high and superior level compared to other materials such as zeolites, and they retain significant regenerative capacity. Except for the MOF with refcode VAJGAR, the regeneration capacity of all other adsorbents exceeds 80%. The substantial degradation in the performance of the MOF with refcode VAJGAR is mainly due to its heightened selectivity towards the impurity gases CO_2_ and H_2_O. This selectivity is likely associated with the robust interactions that CO_2_ and H_2_O can establish with the OMS or hydrophilic locations within the adsorbent’s material structure [60]. The relevant adsorption results for CO_2_ and H_2_O are attached in Table S5. At the same time, detailed adsorption performance indicators for N_2_ and O_2_ adsorption under real gas conditions are listed in Table S4.

## 4. Conclusions

Molecular simulation is a pivotal tool in the development of novel adsorbent materials. However, the vast material space demands substantial computational resources and time. In the quest to discover high-performance adsorbent materials for argon separation from air, this approach still faces significant challenges. In this work, a two-step screening strategy was proposed to facilitate the rapid and effective identification of adsorbent materials for separating argon in air from the CoRE MOF database 2019, which encompasses a staggering 12,020 MOF materials. The research goal was to find materials that could simultaneously adsorb nitrogen and oxygen with high efficiency, thereby enabling argon separation by capturing these gases in the adsorption tower. By analyzing the adsorption properties of randomly selected MOFs, we established a structure–activity relationship between the adsorption capabilities and geometric structures of MOF materials, allowing for a comprehensive evaluation of all adsorption potentials across the entire spectrum of the material space.

Notably, the top 10 MOFs identified in this study outperformed the existing 214 zeolite materials. MOFs with refcode KEVBOE showed a 1.75-fold improvement in performance compared to zeolite materials with refcode AFR. Furthermore, our investigation revealed that zinc (Zn)-based MOFs were the most prevalent, copper (Cu)-based MOFs had the highest concentration of OMSs, and cobalt Co-based MOFs had the lowest proportion of OMSs. However, the presence of OMSs was observed to potentially hinder adsorption efficacy. For the top 10 MOFs, the study also provided a detailed analysis of practical industrial parameters, highlighting the impact of temperature and desorption pressure fluctuations on adsorption performance. For sustainable and large-scale gas separation applications, the interplay between operating conditions, adsorption properties, and economic costs should be comprehensively considered in the future.

## Figures and Tables

**Table 1:** The targeted optimal structural interval, selected by the structure–property relationship.

Descriptor	Unit	Optimal N_2_ Adsorption Interval	Optimal O_2_ Adsorption Interval	Target Optimal Structural Interval
LCD	Å	2.5~17	3~15	3~15
PLD	Å	3~12.5	3~10	3~10
density	g/cm^3^	0.5~2	0.5~2	0.5~2
VSA	m^2^/cm^3^	250~2250	250~2250	250~2250
GSA	m^2^/g	0~2800	0~2250	0~2250
VF	—	0~0.35	0~0.25	0~0.25

**Table 2:** Detailed information on 10 promising MOFs for separating argon from air.

MOF Name	APS (mol/kg, N_2_)	R% (N_2_)	APS (mol/kg, O_2_)	R% (O_2_)	APSA
KEVBOE	1.723	81.25	0.75	83.43	1.29
CUHLUO	1.78	82.45	0.70	84.08	1.24
HUDCEQ	1.83	89.50	0.58	90.56	1.07
cg4012185_si_002	2.01	86.49	0.50	85.56	1.01
GANBAZ01	1.41	85.189	0.61	86.66	0.86
MIXYOJ	1.76	82.74	0.48	84.26	0.84
PETWES	1.65	88.50	0.49	88.25	0.81
VAJGAR	2.56	81.94	0.31	83.47	0.79
PETWIW	1.56	88.16	0.49	88.51	0.77
GANBAZ	1.36	85.45	0.56	86.83	0.76

**Table 3:** Adsorption property of top 10 MOFs selected in this study under more rigorous actual gas circumstances.

MOF Name	S (CO_2_)	S (H_2_O)	APSA	APSA’	Has OMS
KEVBOE	88.14	0.50	1.29	1.24	NO
CUHLUO	55.40	0.41	1.24	1.08	NO
HUDCEQ	34.25	0.50	1.07	0.67	NO
cg4012185_si_002	74.93	0.49	1.01	0.79	YES
GANBAZ01	87.33	0.44	0.86	0.81	NO
MIXYOJ	66.88	3.51	0.84	0.80	NO
PETWES	24.98	0.47	0.81	0.76	YES
VAJGAR	225.09	2.28	0.79	0.03	YES
PETWIW	21.85	0.49	0.77	0.73	YES
GANBAZ	76.17	0.54	0.76	0.76	NO

APSA indicates the performance of MOF adsorbents for the separation of argon in air under ideal gas conditions, and APSA’ represents the performance of MOF adsorbents for separating argon from air under conditions close to real gases.

## Data Availability

All data related to molecular simulation can be seen in https://github.com/xuxuxxy/MOFs_highthroughput-sreening/tree/main/CoREMOF2019 (accessed on 6 March 2025).

## Supplementary Materials

The following supporting information can be downloaded at https://www.mdpi.com/article/10.3390/nano15060412/s1, Figure S1: Similarity analysis of 6 geometric descriptors; Figure S2: (a) Argon adsorption capacity of IRMOF-74-V at 87K derived from GCMC simulations with the parameters and force field set by this study and from the literature; (b) argon adsorption capacity of IRMOF-1 at 87K derived from GCMC simulations with the parameters and force field set by this study and from the literature; Figure S3: The relationship between the number of cycles and the average loading on AHOKIR01; Figure S4: The relationship between the number of cycles and the average loading on BEVRAW; Figure S5: The relationship between the number of cycles and the average loading on BOHGOU; Figure S6: (a) The relationship between APS and the geometric descriptor of 20% MOFs randomly selected in the pre-screened database based on nitrogen, (b) the relationship between the adsorbent performance score (APS) and the geometric descriptor of 20% MOFs randomly selected in the pre-screened database based on oxygen; Figure S7: The impact of open metal sites on the adsorbent performance score (APS); Figure S8: Crystal visualization structure of KEVBOE; Figure S9: Crystal visualization structure of CUHLUO; Figure S10: Crystal visualization structure of HUDCEQ; Figure S11: Crystal visualization structure of cg4012185_si_002; Figure S12: Crystal visualization structure of GANBAZ01; Figure S13: Crystal visualization structure of MIXYOJ; Figure S14: Crystal visualization structure of PETWES; Figure S15: Crystal visualization structure of VAJGAR; Figure S16: Crystal visualization structure of PETWIW; Figure S17: Crystal visualization structure of GANBAZ; Table S1: Lennard–Jones parameters of MOFs; Table S2: Lennard–Jones parameters of Ar, N_2_, O_2_, CO_2_, and H_2_O; Table S3: The metal types of MOFs in the targeted optimal geometric structure range and the situation of OMS; Table S4: Detailed data on the adsorption performance of the top 10 MOFs in real gas conditions; Table S5: Detailed data on the adsorption performance of the top 10 MOFs in real gas conditions. Table S6. The detailed parameters of the T-SNE algorithm.

## Abbreviations

The following abbreviations are used in this manuscript:

APS	adsorbent performance score
APSA	adsorbent performance score for separating argon from air
CIF	Crystallographic Information File
CoRE	computationally ready experimental
GCMC	Grand Canonical Monte Carlo
GSA	geometric surface area
HTCS	high-throughput computational screening
LCD	largest cavity diameter
MOF	metal–organic framework
OMS	existence of open metal site
PLD	pore-limiting diameter
PSA	pressure swing adsorption
UFF	Universal Force Field
VSA	volume surface area
