# Supplementary material for: A Facile Two-Step High-Throughput Screening Strategy of Advanced MOFs for Separating Argon from Air
Source: Nanomaterials (Basel). 2025 Mar 7;15(6):412. doi: 10.3390/nano15060412 (PMC11945806; doi:10.3390/nano15060412)
Supplement: Supplementary file 1 [file nanomaterials-15-00412-s001.zip › nanomaterials-3493310-supplementary.pdf]

A Facile two-step high-throughput screening strategy of advanced MOFs for separating argon from air

Xiaoyi Xu [1], Bingru Xin [1], Zhongde Dai [2], Chong Liu [1], Li Zhou [1], Xu Ji [1,*] and Yiyang Dai [1,*]

[1] School of Chemical Engineering, Sichuan University, Chengdu 610065, China; daiyy@scu.edu.cn
[2] School of Carbon Neutrality Future Technology, Sichuan University, Chengdu 610065, China; zhong-de.dai@scu.edu.cn
* Corresponding Authors: Xu Ji: jixu@scu.edu.cn; Yiyang Dai: daiyy@scu.edu.cn

Catalogue

Table S1. Lennard–Jones parameters of MOFs..2
Table S2. Lennard–Jones parameters of Ar, N_2, O_2, CO_2, H_2O[1]...3
Table S3. The metal types of MOFs in the targeted optimal geometric structure range and the situation of open metal sites (OMSs). 3
Table S4. Detailed data on the adsorption performance of the top 10 MOFs in real gas conditions...4
Table S5. Detailed data on the adsorption performance of the top 10 MOFs in real gas conditions...4
Table S6. The detailed parameters of the T-SNE algorithm 5
Figure S1. Similarity analysis of 6 geometric descriptors...6
Figure S2..6
Figure S3. The relationship between the number of cycles and the average loading on AHOKIR01 7
Figure S4. The relationship between the number of cycles and the average loading on BEVRAW 8
Figure S5. The relationship between the number of cycles and the average loading on BOHGOU 8
Figure S6. (a)The relationship between the APS and geometric descriptor of 20% MOFs randomly selected in the pre-screened database based on nitrogen. 9
Figure S6. (b)The relationship between the adsorbent performance score (APS) and geometric descriptor of 20% MOFs randomly selected in the pre-screened database based on oxygen. 9
Figure S7. The impact of open metal sites on the adsorbent performance scorev(APS) 10
Figure S8. Crystal visualization structure of KEVBOE...10
Figure S9. Crystal visualization structure of CUHLUO 11
Figure S10. Crystal visualization structure of HUDCEQ 11
Figure S11. Crystal visualization structure of cg4012185_si_002 12
Figure S12. Crystal visualization structure of GANBAZ01...12
Figure S13. Crystal visualization structure of MIXYOJ 13
Figure S14. Crystal visualization structure of PETWES 13
Figure S15. Crystal visualization structure of VAJGAR 14

Figure S16. Crystal visualization structure of PETWIW..14
Figure S17. Crystal visualization structure of GANBAZ 15
References..15

Table S1. Lennard–Jones parameters of MOFs.

Atom	$\varepsilon/k_b(K)$	σ(Å)	Atom	$\varepsilon/k_b(K)$	σ(Å)	Atom	$\varepsilon/k_b(K)$	σ(Å)
Ac_	16.6	3.1	Ge_	190.69	3.81	Po_	163.52	4.2
Ag_	18.11	2.8	Gd_	4.53	3	Pr_	5.03	3.21
Al_	254.09	4.01	H_	22.14	2.57	Pt_	40.25	2.45
Am_	7.04	3.01	Hf_	36.23	2.8	Pu_	8.05	3.05
Ar_	93.08	3.45	Hg_	193.71	2.41	Ra_	203.27	3.28
As_	155.47	3.77	Ho_	3.52	3.04	Rb_	20.13	3.67
At_	142.89	4.23	I_	170.57	4.01	Re_	33.21	2.63
Au_	19.62	2.93	In_	301.39	3.98	Rh_	26.67	2.61
B_	90.57	3.64	Ir_	36.73	2.53	Rn_	124.78	4.25
Ba_	183.15	3.3	K_	17.61	3.4	Ru_	28.18	2.64
Be_	42.77	2.45	Kr_	110.69	3.69	S_	137.86	3.59
Bi_	260.63	3.89	La_	8.55	3.14	Sb_	225.91	3.94
Bk_	6.54	2.97	Li_	12.58	2.18	Sc_	9.56	2.94
Br_	126.29	3.73	Lu_	20.63	3.24	Se_	146.42	3.75
C_	52.8409	3.43	Lr_	5.53	2.88	Si_	202.27	3.83
Ca_	119.75	3.03	Md_	5.53	2.92	Sm_	4.03	3.14
Cd_	114.72	2.54	Mg_	55.85	2.69	Sn_	285.28	3.91
Ce_	6.54	3.17	Mn_	6.54	2.64	Sr_	118.24	3.24
Cf_	6.54	2.95	Mo_	28.18	2.72	Ta_	40.75	2.82
Cl_	114.21	3.52	N_	34.7214	3.26	Tb_	3.52	3.07
Cm_	6.54	2.96	Na_	15.09	2.66	Tc_	24.15	2.67
Co_	7.04	2.56	Ne_	21.13	2.66	Te_	200.25	3.98
Cr_	7.55	2.69	Nb_	29.69	2.82	Th_	13.08	3.03
Cu_	2.52	3.11	Nd_	5.03	3.18	Ti_	8.55	2.83
Cs_	22.64	4.02	No_	5.53	2.89	TI_	342.14	3.87
Dy_	3.52	3.05	Ni_	7.55	2.52	Tm_	3.02	3.01
Eu_	4.03	3.11	Np_	9.56	3.05	U_	11.07	3.02
Er_	3.52	3.02	O_	30.1948	3.118	V_	8.05	2.8
Es_	6.04	2.94	Os_	18.62	2.78	W_	33.71	2.73
F_	25.16	3	P_	153.46	3.69	Xe_	167.04	3.92
Fe_	6.54	2.59	Pa_	11.07	3.05	Y_	36.23	2.98
Fm_	6.04	2.93	Pb_	333.59	3.83	Yb_	114.72	2.99
Fr_	25.16	4.37	Pd_	24.15	2.58	Zn_	62.39	2.46
Ga_	208.81	3.9	Pm_	4.53	3.16	Zr_	34.72	2.78

Table S2. Lennard–Jones parameters of Ar, N_2, O_2, CO_2, and H_2O [1].

Atom	$\varepsilon/k_b(K)$	σ(Å)
Ar	124.1	3.42
N_n2	38.298	3.31
O_o2	49	3.02
O_co2	79	3.05
C_co2	27	2.8
O_tip4p_2005	93.20	3.159
H_tip4p_2005	0	0
M_tip4p_2005	0	0

Table S3. The metal types of the MOFs in the targeted optimal geometric structure range and the situation of open metal sites (OMSs).

Metal type	Number of MOFs	Number of MOFs with OMS
Zn	935	544
Cu	610	469
Cd	474	226
Co	381	183
Mn	238	151
Ni	197	117
Ag	177	156
Eu	124	92
Fe	124	35
Tb	106	80
Gd	92	71
In	86	11
Nd	85	59
La	83	47
Mg	76	45
V	62	26
Sm	61	47
Dy	59	46
Na	55	41
Pr	50	33

Table S4. Detailed data on the adsorption performance of the top 10 MOFs in real gas conditions.

MOF name	APS′(mol/kg, N_2)	R%′(N_2)	APS′(mol/kg, O_2)	R%′(O_2)	APSA	APSA′
KEVBOE	1.63	82.10	0.76	84.26	1.29	1.24
CUHLUO	1.63	82.52	0.66	84.16	1.24	1.08
HUDCEQ	1.57	87.80	0.43	88.14	1.07	0.67
cg4012185 _si_002	1.70	78.75	0.47	80.39	1.01	0.79
GANBAZ 01	1.34	84.66	0.61	86.35	0.86	0.81
MIXYOJ	1.67	82.21	0.48	83.52	0.84	0.80
PETWES	1.58	88.36	0.48	88.45	0.81	0.76
VAJGAR	0.30	65.55	0.08	70.68	0.79	0.03
PETWIW	1.51	88.26	0.49	88.60	0.77	0.73
GANBAZ	1.34	85.76	0.57	87.12	0.76	0.76

APS′ and R%′ indicate the adsorbent performance score and the recovery of MOF adsorbents for which the target adsorbate is nitrogen or oxygen under conditions close to real gases, APSA indicates the performance of MOF adsorbents for the separation of argon in air under ideal gas conditions, and APSA′ represents the performance of MOF adsorbents for separating argon from air under conditions close to real gases.

Table S5. Detailed data on the adsorption performance of the top 10 MOFs in real gas conditions.

MOF name	S(CO_2)	S(H_2O)
KEVBOE	88.14	0.50
CUHLUO	55.40	0.41
HUDCEQ	34.25	0.50
cg4012185_si_00 2	74.93	0.49
GANBAZ01	87.33	0.44
MIXYOJ	66.88	3.51
PETWES	24.98	0.47
VAJGAR	225.09	2.28
PETWIW	21.85	0.49
GANBAZ	76.17	0.54

S(CO_2) and S(H_2O) indicate the selectivity of CO_2 and H_2O.

Table S6. The detailed parameters of the T-SNE algorithm.

n_components	2
perplexity	15
learning_rate	100
n_iter	1000

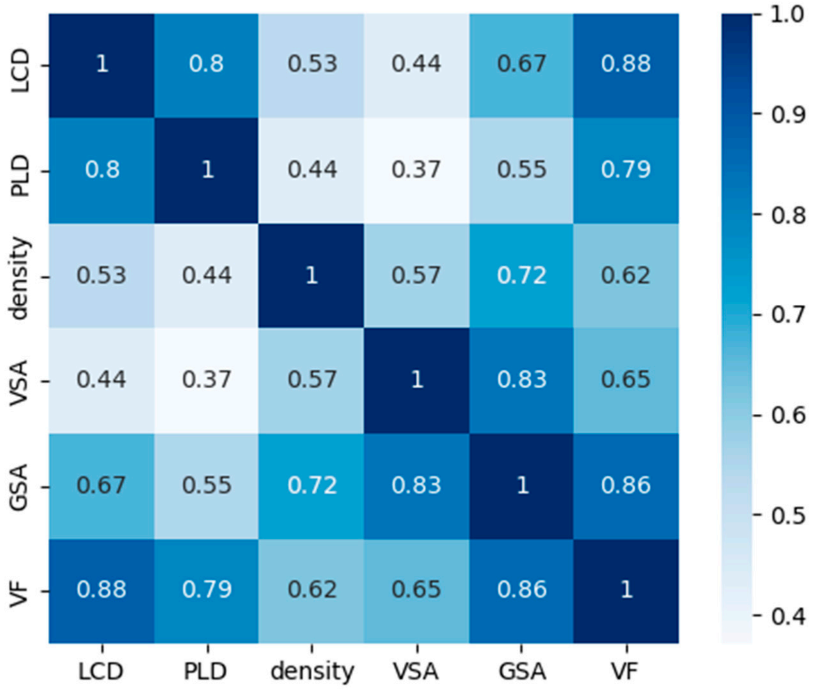

Figure S1. Similarity analysis of 6 geometric descriptors.

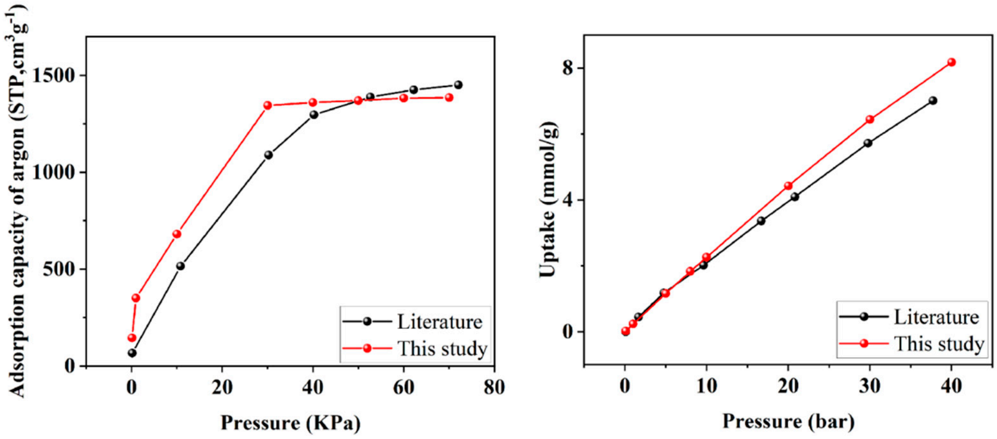

Figure S2. (**a**) Argon adsorption capacity of IRMOF-74-V at 87K derived from GCMC simulations with the parameters and force field set by this study and from the literature [2]; (**b**) argon adsorption capacity of IRMOF-1 at 87K derived from GCMC simulations with the parameters and force field set by this study and from the literature [3].

From both figures, it can be observed that the results from this study are in good agreement with the data in the literature, thereby validating the reasonableness of the parameters and force field set for the GCMC simulation in this study.

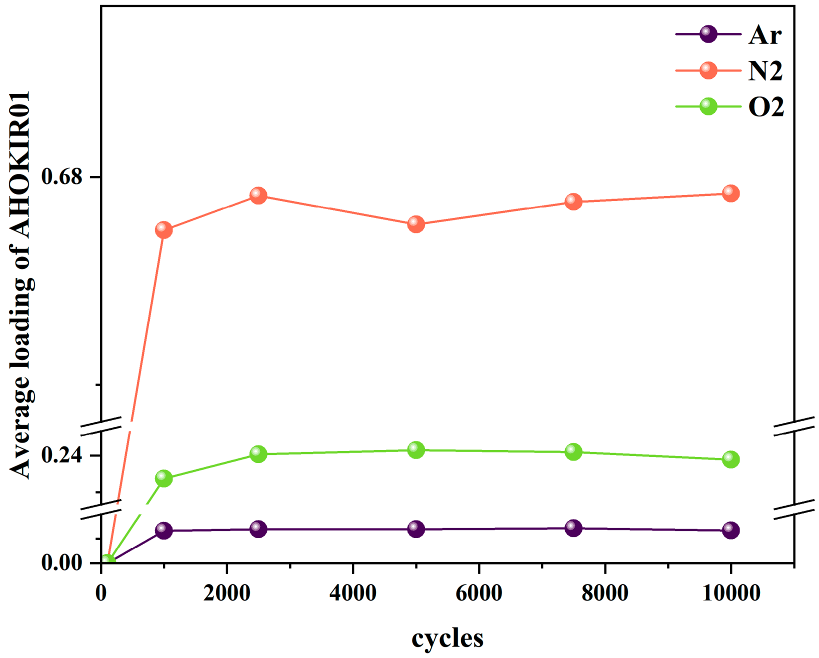

Figure S3. The relationship between the number of cycles and the average loading on AHOKIR01.

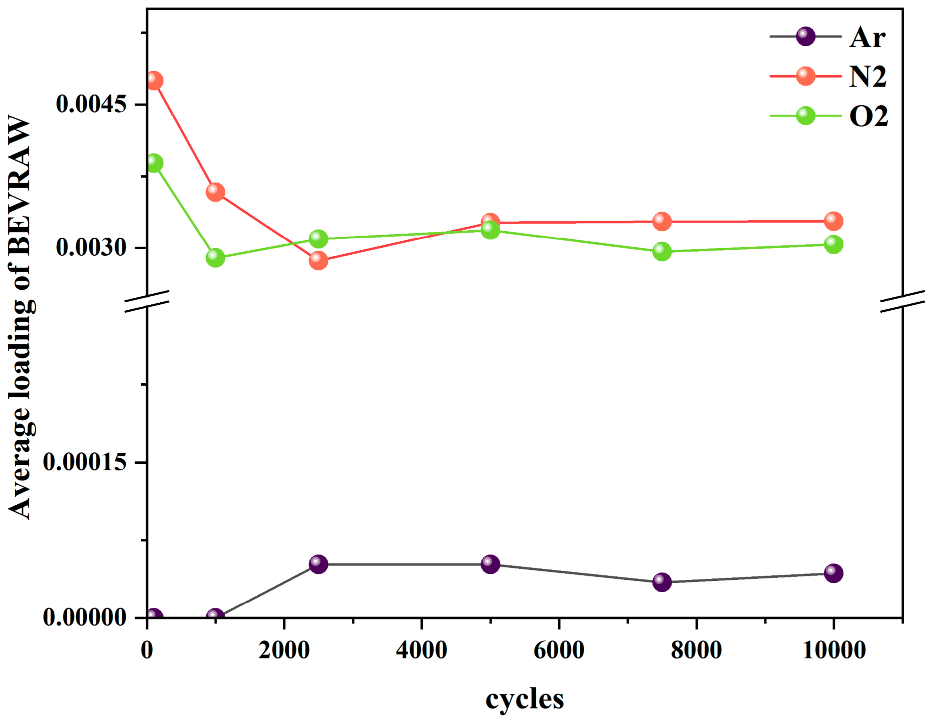

Figure S4. The relationship between the number of cycles and the average loading on BEVRAW.

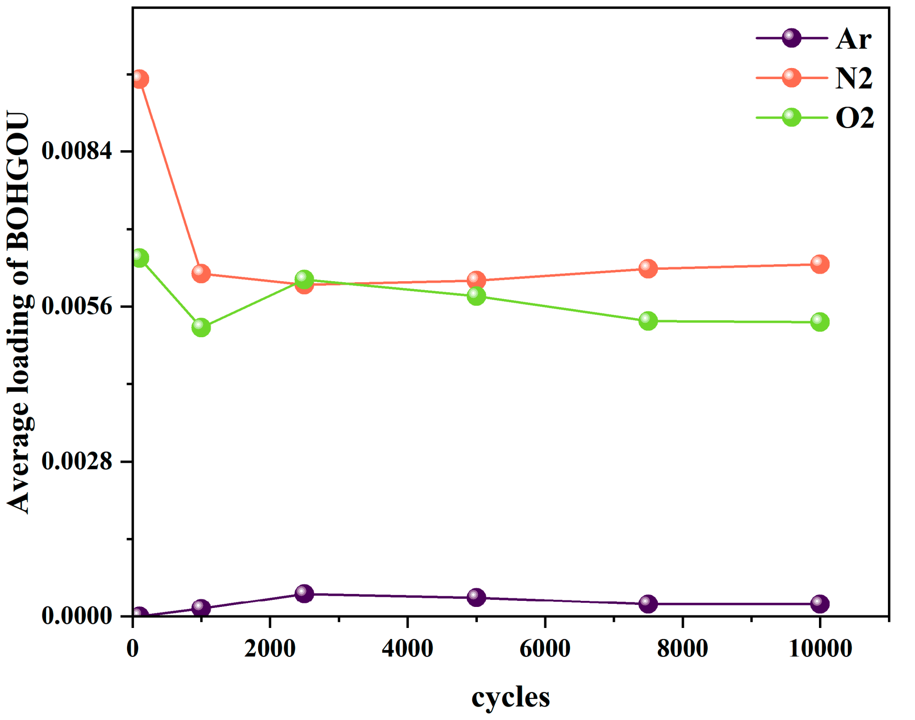

Figure S5. The relationship between the number of cycles and the average loading on BOHGOU.

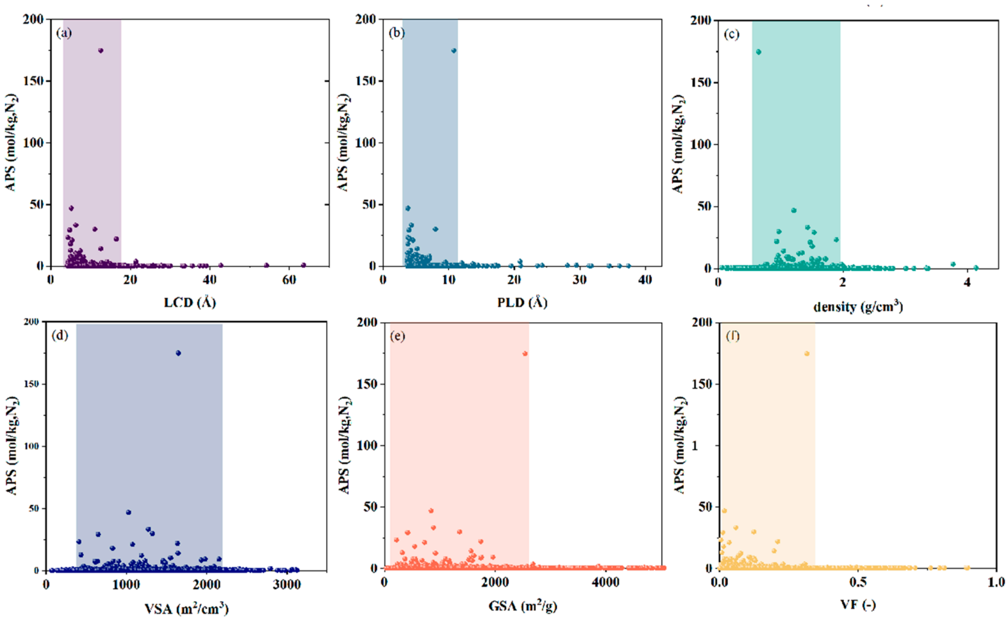

Figure S6. (**a**) The relationship between the APS and geometric descriptor of 20% MOFs randomly selected in the pre-screened database based on nitrogen.

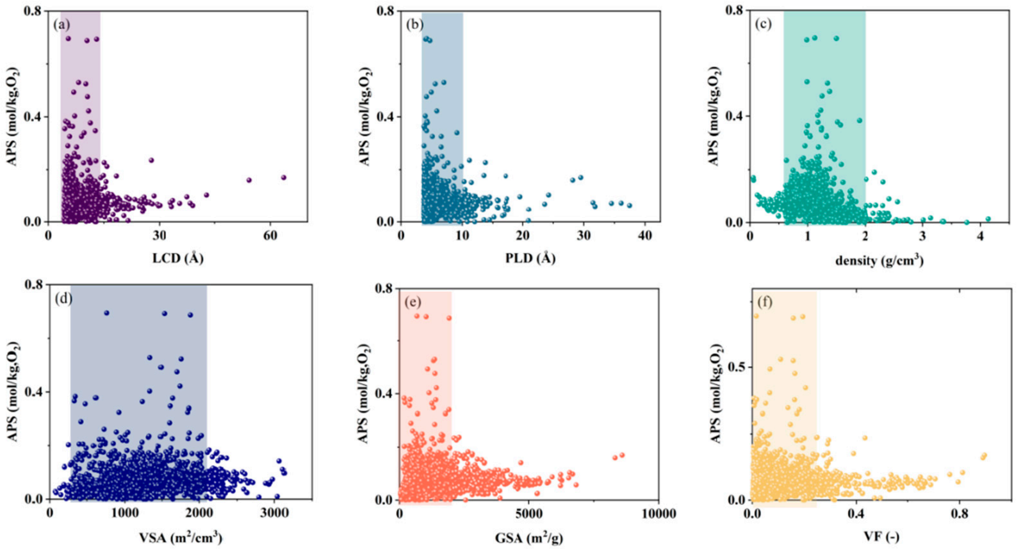

Figure S6. (**b**) The relationship between the adsorbent performance score (APS) and geometric descriptor of 20% MOFs randomly selected in the pre-screened database based on oxygen.

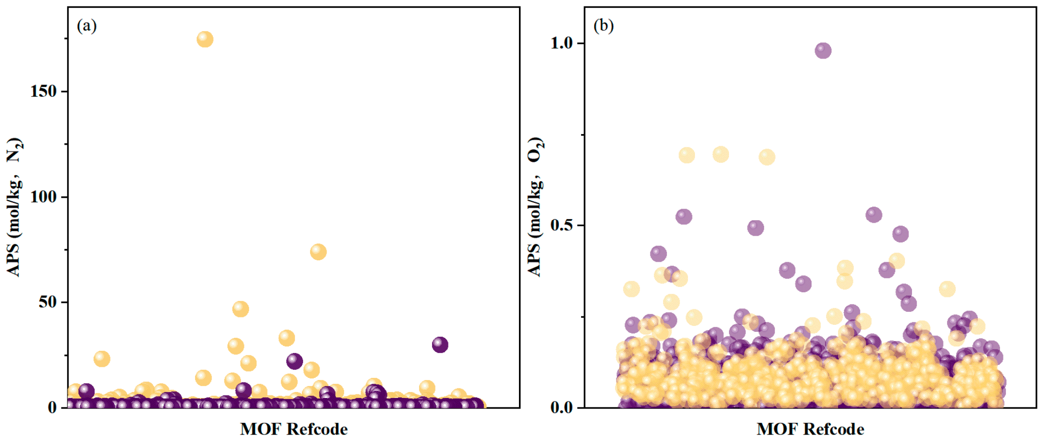

Figure S7. The impact of open metal sites on the adsorbent performance score (APS), where purple spheres represent the presence of open metal sites and yellow spheres indicate the absence of open metal sites.

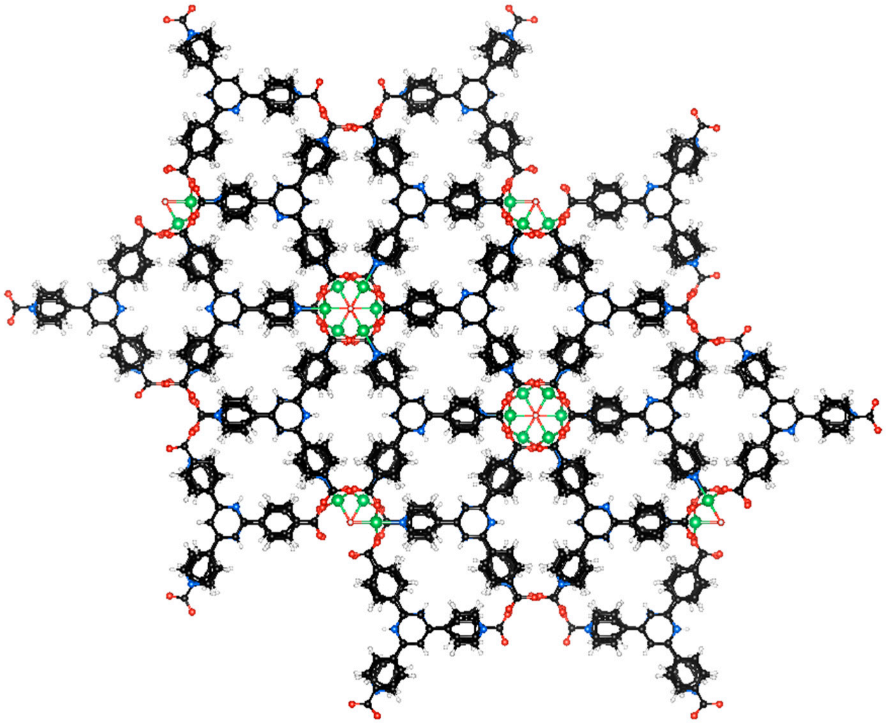

Figure S8. Crystal visualization structure of KEVBOE.

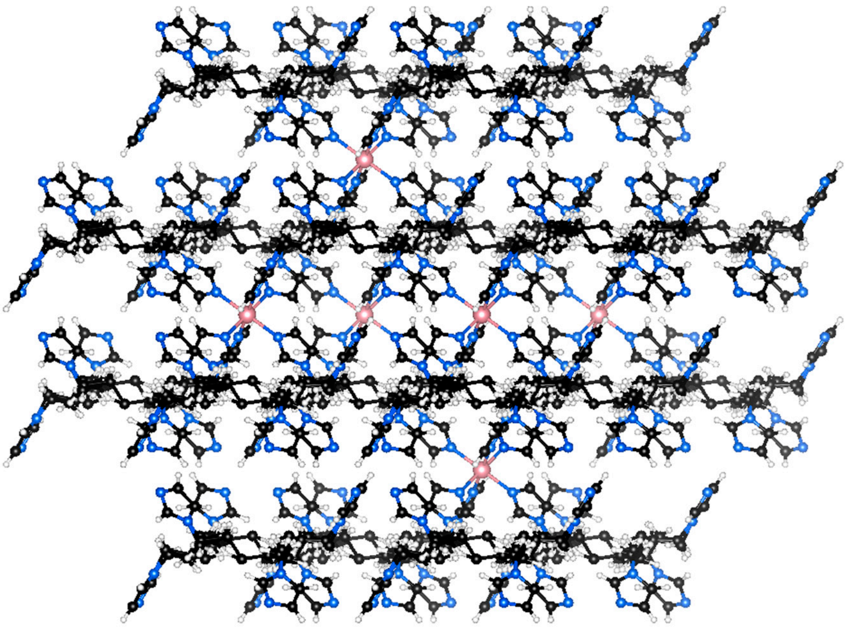

Figure S9. Crystal visualization structure of CUHLUO.

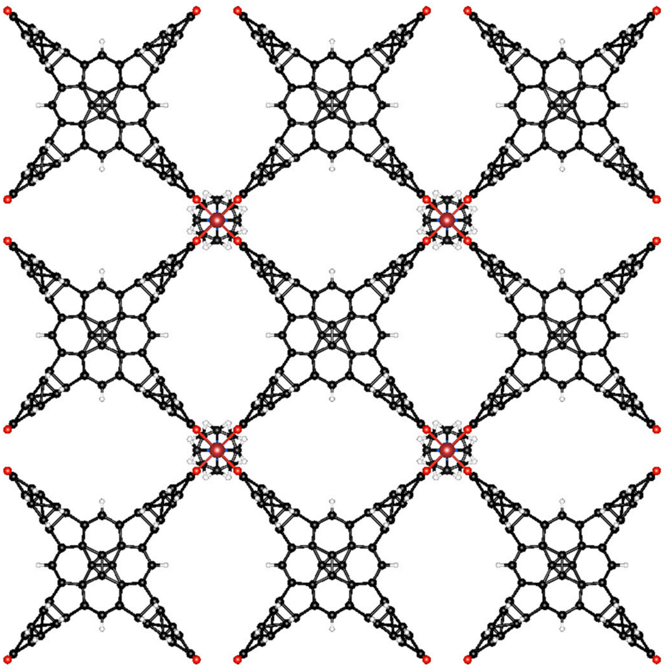

Figure S10. Crystal visualization structure of HUDCEQ.

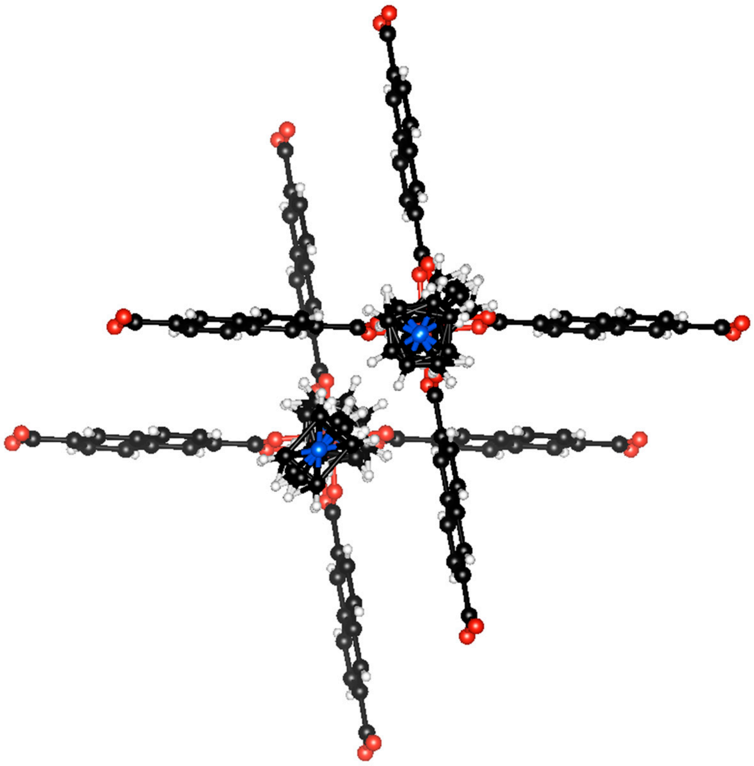

Figure S11. Crystal visualization structure of cg4012185_si_002.

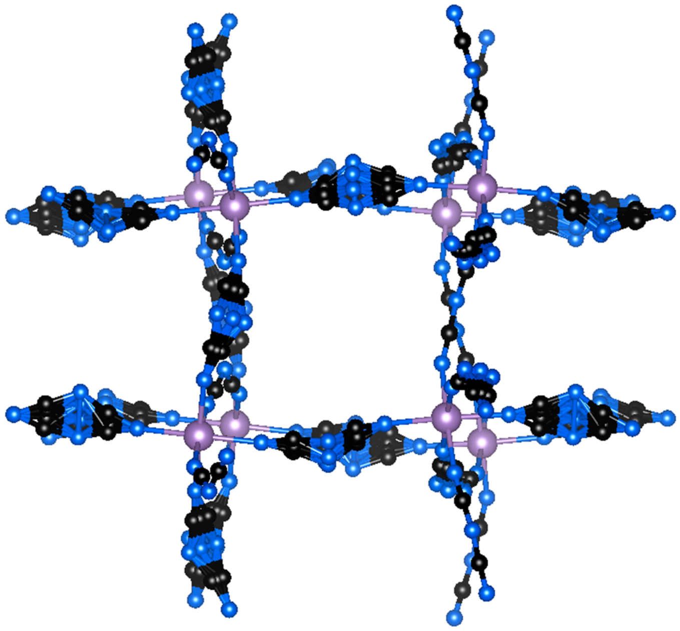

Figure S12. Crystal visualization structure of GANBAZ01.

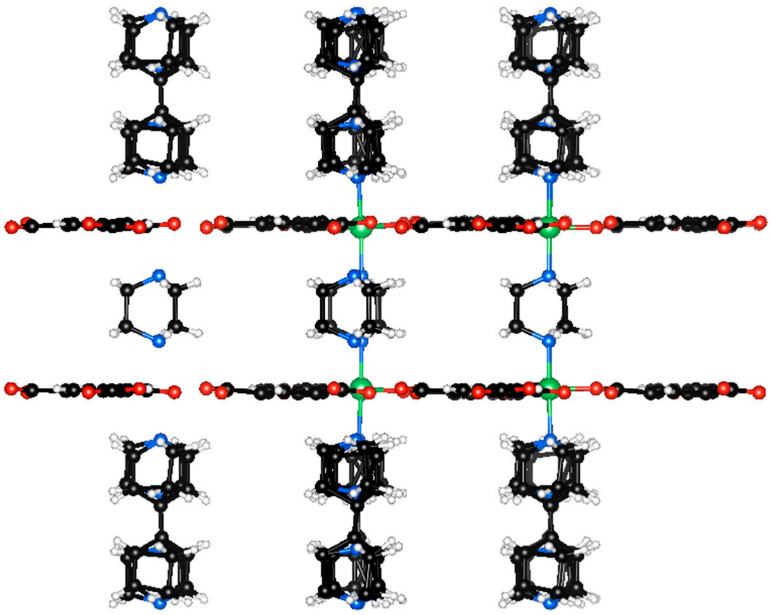

Figure S13. Crystal visualization structure of MIXYOJ.

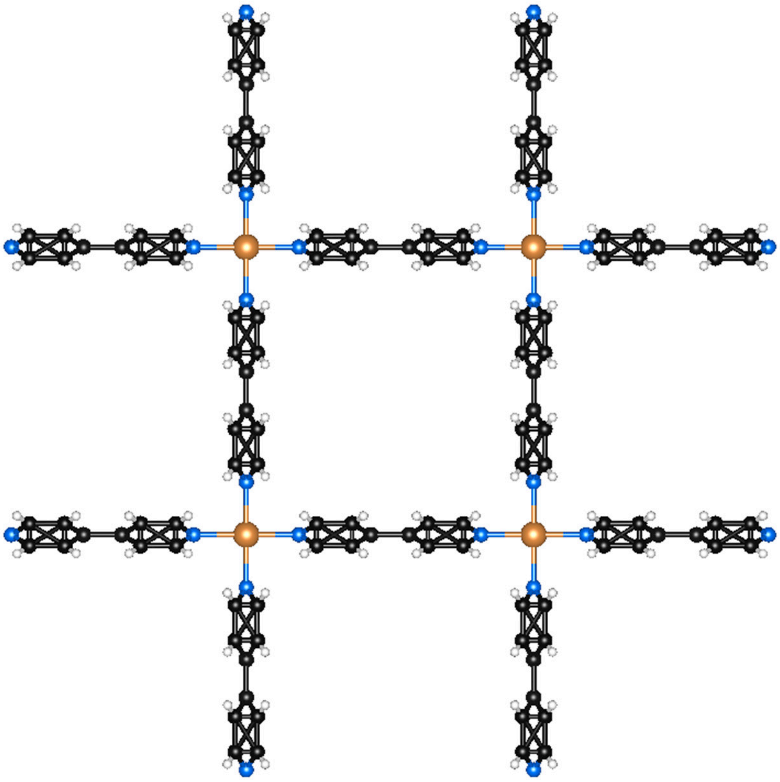

Figure S14. Crystal visualization structure of PETWES.

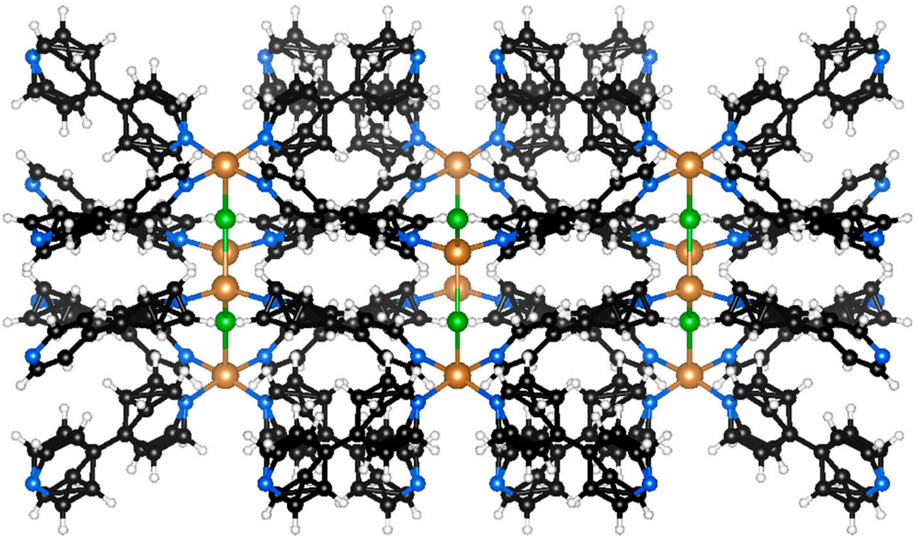

Figure S15. Crystal visualization structure of VAJGAR.

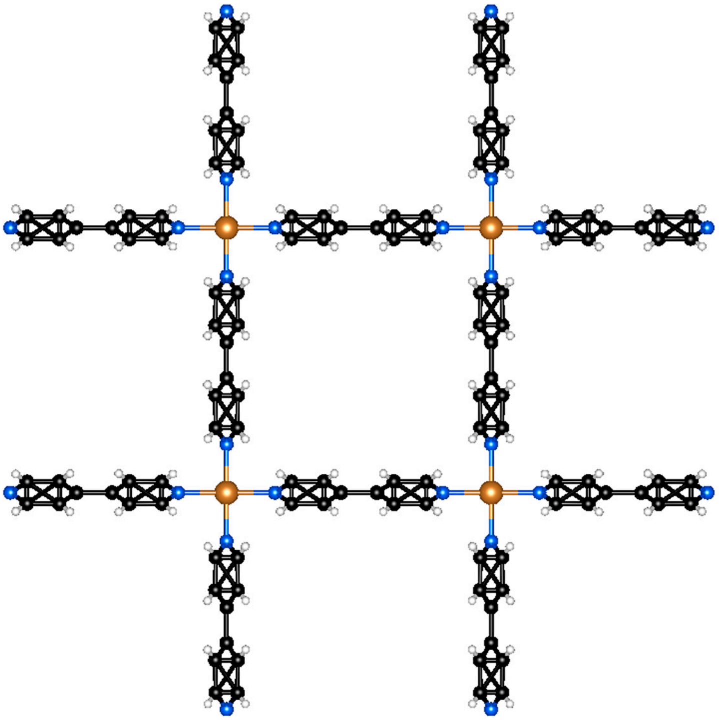

Figure S16. Crystal visualization structure of PETWIW.

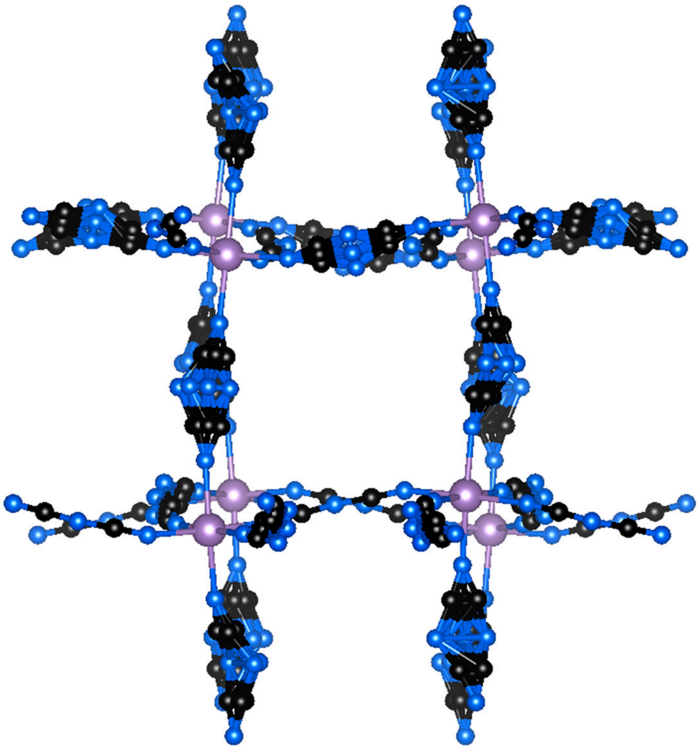

Figure S17. Crystal visualization structure of GANBAZ.

References

1. Míguez, J.M.; González-Salgado, D.; Legido, J.L.; Piñeiro, M.M. Calculation of Interfacial Properties Using Molecular Simulation with the Reaction Field Method: Results for Different Water Models. *The Journal of Chemical Physics* **2010**, *132*, 184102, doi:10.1063/1.3422528.
2. Furukawa, H.; Cordova, K.E.; O'Keeffe, M.; Yaghi, O.M. The Chemistry and Applications of Metal-Organic Frameworks. *Science* **2013**, *341*, 1230444, doi:10.1126/science.1230444.
3. Parkes, M.V.; Staiger, C.L.; Iv, J.J.P.; Allendorf, M.D.; Greathouse, J.A. Screening Metal–Organic Frameworks for Selective Noble Gas Adsorption in Air: Effect of Pore Size and Framework Topology. *Phys. Chem. Chem. Phys.* **2013**, *15*, 9093–9106, doi:10.1039/C3CP50774B.
